# The Impact of Consumers’ Choice Deferral Behavior on Their Intertemporal Choice Preference

**DOI:** 10.3389/fpsyg.2021.555150

**Published:** 2021-05-06

**Authors:** He-Lin Wei, Chen-Ying Hai, Shao-Ying Zhu, Bei Lyu

**Affiliations:** ^1^Business School, Guangxi University, Nanning, China; ^2^School of Economics and Management, Huaibei Normal University, Huaibei, China; ^3^Business School, Henan University, Kaifeng, China; ^4^Chinese Graduate School, Panyapiwat Institute of management, Nonthaburi, Thailand; ^5^Leeds University Business School, University of Leeds, Leeds, United Kingdom

**Keywords:** choice deferral, intertemporal choice, hopefulness, information integrity, impact

## Abstract

The purpose of this study is to explore the influence of consumers’ choice deferral behavior on their intertemporal choice preference. The empirical study shows that consumers’ choice deferral behavior can significantly affect their intertemporal decision preference through the level of hopefulness. Compared with non-choice deferral behavior, choice deferral behavior can improve the level of consumers’ sense of hopefulness, which then makes them prefer larger-longer interests in intertemporal decision-making. The effect of consumers’ sense of hopefulness on their intertemporal choice preference is moderated by their perceived information integrity. When the perceived information integrity is low, the effect of hopefulness on intertemporal decision preference will be enhanced, but when the perceived information integrity is high, the effect of hopefulness on intertemporal decision preference will not be affected. In addition, the theoretical and practical significance of this study and the prospect of future research are also discussed.

## Introduction

Due to the variety of products, the uncertainty of consumers’ preferences, and other reasons, consumers often find it difficult to make decisions, which leads them to give up making purchase decisions at present and choose to postpone making decisions later (Li and Xie, 2012). With the increasing richness of product information, shopping decisions become more complex, and consumers’ choice deferral behavior becomes more common and prominent (Sun et al., 2019). This not only wastes consumers’ decision-making time and brings poor shopping experience and more negative emotions to consumers, but also is not conducive to immediate product sales of merchants (Sagi and Friedland, 2007; Liu N. et al., 2017; Lu and Wang, 2018). When shopping, consumers often encounter intertemporal decision-making problems. For example, when a new product is released, do you choose to buy it at a higher price and start to experience the latest product as soon as possible (shorter-smaller benefit, SS), or prefer to wait for a period of time before the price drops. At that time, although the product is not the latest, the price is more favorable (longer-larger benefit, LL), which involves consumers’ choice of interests at different time points, as well as the profit plans of businesses at different time points. Some studies have found that consumers’ choice deferral behavior can affect their future shopping decision preference (Li and Fu, 2006). Then, whether and how consumers’ choice deferral behavior can affect their intertemporal choice preference becomes the main research problem of this paper. This problem is not only related to how consumers deal with the intertemporal decision-making in shopping more rationally, but also affects how enterprises estimate consumers’ preferences in intertemporal decision-making by considering their choice deferral behavior, and design corresponding marketing schemes at different time points, so as to improve marketing efficiency, achieve accurate marketing, and finally obtain greater profits.

Individual choice deferral behavior and intertemporal choice preference are both hot topics in recent years, but few scholars consider the relationship between them (White et al., 2015; Leonardo and Severine, 2017; Keskin, 2020; Martin et al., 2020). Intertemporal decision-making reflects how people view the value of things at different time points (Liang and Liu, 2011), and the individual’s perception of their own time adequacy can affect their intertemporal choice preference (Li et al., 2016). The choice deferral behavior delays the decision-making time and increases the decision-making time (Li and Tu, 2011), therefore, it is speculated that choice deferral behavior can affect consumers’ intertemporal choice preference by influencing consumers’ perception of time distance of options. Most scholars believe that consumers’ decision-making efficiency will be reduced if they do not make immediate consumption due to choice deferral behavior, and the final purchase possibility will also be affected, which is not conducive to merchants’ immediate sales of products (Li and Zhang, 2010). Therefore, most scholars are committed to finding out the factors that affect consumers’ choice deferral behavior, in order to reduce consumers’ choice deferral behavior (Pethtel and Chen, 2013; Mourali et al., 2018; Lin et al., 2020). However, consumers still have the possibility to buy after they choose to postpone their purchase. With the development of the commodity economy, products are updated faster and marketing models are more diverse. Consumers will still encounter many similar problems of delayed choice in the future. Therefore, it is very meaningful to study the aftereffect of delayed choice. This study focuses on exploring the influence path of choice deferral behavior on subsequent intertemporal choice preference, which enriches the related research of choice deferral behavior and intertemporal choice preference, has certain theoretical value, and also provides some reference for the marketing practice of enterprises.

To sum up, based on the existing research results, this paper puts forward research hypotheses, constructs a model of the impact of consumers’ choice deferral behavior on their intertemporal choice preference, and attempts to explore the relationship between the two, which is verified by empirical analysis. Finally, by comparing this study with previous studies, the theoretical contribution, practical significance, and research defects of this paper are clarified, and the future research direction is prospected.

### Intertemporal Choice Preference

Intertemporal choice preference is a hot topic in behavioral decision-making and related fields, which refers to the decision-making subject balancing the options of different times and different interests, and then making various judgments and choices (Frederick et al., 2002). For the convenience of research, scholars tend to simplify this problem into two kinds of options, one is the shorter-smaller interests (SS), the other is the LL (Liu et al., 2015). This study is to refer to this research method, by understanding consumers’ preferences for these two kinds of options, to judge their intertemporal choice preferences. There is abundant research on antecedents influencing intertemporal choice preference, which can be summarized into three categories. The first is the personal factors of decision makers. For example, an individual’s preference for money will affect his intertemporal choice preference. The more he likes money, the more he prefers larger long-term interests (Yang et al., 2018); the second type is the characteristics of decision-making tasks. For example, in the face of decisions in different time orientations (such as past or future), decision makers have different intertemporal choice preferences (Zhuang et al., 2017). In addition, different descriptions of the same option will also affect individual intertemporal decision-making preferences, which is also called “framing effect” (Read et al., 2005); the third type is the background characteristics of decision-making. For example, the tense social rhythm makes people aware of time poverty, so they are more short-sighted in intertemporal decision-making, and prefer short-term smaller interests (Li et al., 2016). Relatively speaking, few studies focus on the length of time spent on making intertemporal choice, such as whether to delay the choice. A delayed choice makes consumers make decisions later, so that they have more time to make decisions on shorter-smaller interests (SS) and LL. The problem of this paper is how consumers’ intertemporal choice preferences change after they make a delayed choice. According to previous studies, the time and amount of intertemporal decision-making options can affect individual intertemporal choice preference (Li and Wang, 2014). Therefore, in the experiment, the time and amount of intertemporal decision-making options are used as control variables to avoid their impact on the whole experiment.

### Choice Deferral Behavior

**FIGURE 1:**
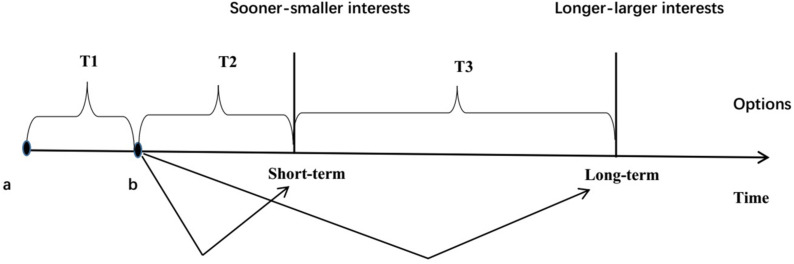
Intertemporal decision making in the case of choice deferral behavior.

**FIGURE 2:**
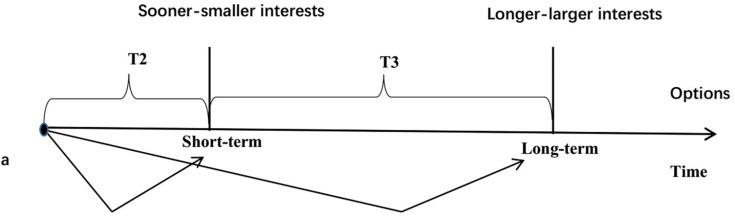
Intertemporal decision making in the case of non-choice deferral behavior.

Choice deferral behavior is one of many decision-making behaviors, which means that a decision-maker does not make a choice at the time a decision is required, but postpones decisions and chooses later (Anderson, 2003) — in other words, there is a specific time interval between the need to make a decision and real decision-making. The opposite of choice deferral behavior is non-choice deferral behavior, that is, to make a decision when it is necessary to make a choice, not to refuse the choice and not to delay the choice time. Previous studies on choice deferral behavior focused on factors affecting choice deferral behavior, which we divided into three aspects: decision task’s attributes (e.g., selection set size, Liu T. et al., 2017), decision-maker’s personal factors (e.g., the scope of decision-making power, Li and Jiang, 2019), and environmental factors of decision-making (e.g., time pressure, Lu and Wang, 2018). Choice deferral behavior can affect the decision-maker’s emotions, their decision-making process, and the decision results (Li and Fu, 2006), etc. For example, research has found that when a decision-maker has no definite preference and must choose a given product, the effect of compromise is significantly weakened if a choice deferral option is added (Dhar and Simonson, 2003). When analyzing the influence of choice deferral behavior on decision-maker emotions, research has found that choice deferral behavior reduces decision-makers’ negative emotions when they experience emotional trade-offs (Luce, 1998). These achievements provide strong theoretical support for studying the relationship between choice deferral behavior and intertemporal choice preference. Next, Figures 1, 2 are used to illustrate the relationship between choice deferral and intertemporal choice preference. As shown in Figure 1, the intertemporal choice preference in the case of choice deferral behavior means that consumers do not make a decision at time A when they need to make a decision, but delay the intertemporal decision at time B after time T1. The options are shorter-smaller interests (SS) after time T2 (for example, buying A after 1 day costs 10 Yuan) and LL after time T2 + T3 (for example, buying A after 5 days costs 8 Yuan). As shown in Figure 2, the intertemporal decision preference in the case of non-choice deferral behavior means that consumers make intertemporal decision at a time point, and the option is still the shorter-smaller interests (SS) after T2 (for example, buying A after 1 day costs 10 Yuan) and the LL after T2 + T3 (for example, buying A after 5 days costs 8 Yuan). This study is based on the non-choice deferral behavior as a reference to study how consumers’ intertemporal choice preference is under the choice deferral behavior.

### Perceived Information Integrity

Perceived information integrity refers to decision-makers’ subjective perception of the integrity of information required to make decisions. When consumers make purchasing decisions, they judge according to the information they have. Many scholars divide the attributes of information into quantity and quality (Anish et al., 2011), and mostly study information’s impact on decision-making from these two aspects. However, in this era of big data, it is impossible for individuals to grasp all of the available information, and their information processing abilities are different, thus their demand for the quantity and quality of information is different. Therefore, this study considers an individual’s demand for information from a subjective perspective. From the subjective point of view, consumers have different evaluation criteria for information integrity. In order to more accurately understand the impact of consumers’ perception of information integrity on their shopping decisions, we must unify consumers’ evaluation criteria for information integrity. Nevertheless, scholars have different opinions on information integrity. In studies on individual investors’ information acquisition behavior in the context of big data, some scholars delineated that information integrity should be judged from two aspects—comprehensiveness and integrity (Wang et al., 2018). One scholar studied the online shopping reviews’ utility and believed that measures for information integrity should be quantity and comprehensiveness (Zhu et al., 2017). Since this paper mainly studies consumer decision-making while shopping, which is closely related to the latter study’s research on the usefulness of online shopping reviews, we refer to this study’s evaluation criteria to measure information integrity in terms of quantity and comprehensiveness.

### Hopefulness

Hopefulness specifically impacts decision-making. Previous studies on hopefulness have focused on people’s mental health, healthcare, education, and other factors, while this paper focuses on the role of hopefulness in consumer behavior. Scholars have different understandings of hopefulness, which can be roughly divided into three categories. In the first category, some scholars classified hopefulness as an emotion, believing that it is an emotion generated during a difficult situation (Miller and Powers, 1986; Liu and Huang, 2013). Second, some scholars classified hopefulness in a cognitive category, believing that hopefulness refers to one’s thoughts about their own abilities and future development status (Breznitz, 1986; Morse and Doberneck, 1995). Third, some scholars defined hopefulness as a combination of the first two categories (Staats, 1989; Weinberg et al., 2016). Among them, Snyder et al.’s definition of hopefulness belongs in the third category; their definition is well recognized by the academic community. They believe that hopefulness is a kind of positive emotions that are brought about by the interaction of dynamic thought (the power to achieve goals) and path thought (the way to achieve goals) (Chang, 2015). Here, we use this definition of hopefulness to study the effect of a consumer’s level of hopefulness on choice deferral behavior and intertemporal choice preference.

## Hypotheses

Choice deferral behavior and intertemporal choice preference are related to the time distance. Choice deferral behavior can directly increase decision-making time, while intertemporal decision making is choosing different interests at different time points. The construal level theory includes an individual’s perception mechanism of things at different time distances. It holds that individuals explain choices at a high-level interpretation of things at a long time distance, that is, the individual experience looks at things from an abstract, holistic, essential, and core point of view; the individual has a low level of interpretation of things at a short time distance, that is, the individual experience looks at things from a specific, superficial, and situational point of view (Jiang and He, 2017). In the process of intertemporal choice making, the perceived difference of time distance significantly affects the individual’s choice tendency (Suo et al., 2014), the perception of individual’s time adequacy can affect their intertemporal choice preference (Li et al., 2016). Another study shows that people basically prefer benefits in the near future (Thaler and Shefrin, 1981), but people prefer LL if the benefit time of the two options is extended simultaneously (Chen and He, 2011). Therefore, we speculate that choice deferral behavior can increase consumers’ perception of time distance by increasing decision-making time. According to the construal level theory and previous studies, we conclude that choice deferral behavior can affect consumers’ perception of time distance of options by increasing decision-making time and making consumers think that the time distance of options becomes longer, which leads them to pay more attention to the nature of things and the overall interests, and choose the option with LL in intertemporal decision-making. In contrast, under the condition of non-choice deferral behavior, consumers perceive a shorter time distance, which leads them to pay more attention to partial interests and the surface of things, so they choose the option with shorter-smaller interests (SS). Based on these facts, hypothesis H1 is proposed.

H1: Consumers’ choice deferral behavior can significantly impact intertemporal choice preference. Non-choice deferral behavior can cause consumers to prefer sooner-smaller interests (SS); choice deferral behavior can cause consumers to prefer LL.

Hopefulness is a kind of positive emotion, which includes two dimensions: dynamic thought (the power to achieve goals) and path thought (the way to achieve goals) (Snyder et al., 1991). Choice deferral behavior can affect the decision-maker’s emotions, information extraction process, and decision-making results (Li and Fu, 2006). Decision-makers’ choice deferral behavior can be attributed to have choice difficulties (Zhuang et al., 2017), and they defer choice in order to better understand a situation or gain more time to make a decision (Leonardo and Severine, 2017), such as facing non-essential products, or they do not have enough information. To make a delayed choice is to better understand the relevant situation or to get more time to make decisions. At this time, decision-makers have the motivation to optimize decision-making, and will actively think about various ways to solve problems, which reflects dynamic thought and the thought pattern of hopefulness. In addition, when the emotional trade-off is difficult, choice deferral behavior can reduce the negative emotions of decision-makers (Luce, 1998). Decision makers may foresee that they will regret making a certain decision, so they want to solve this problem by delaying the choice (Anderson, 2003). These studies show that choice deferral behavior can make decision-makers’ emotions tend to be positive, and the hopefulness is a positive emotion. In contrast, consumers do not defer choice, either because they have enough product information or because they need to buy products urgently. At this time, consumers will not have more motivation or find more ways to optimize their choice, and their hopefulness will be low. However, when consumers do not have enough information or the product is not a necessity, and can only make a decision immediately when they need to make a decision, consumers will not have more opportunities and time to weigh the options, so as to reduce the motivation of optimal decision-making, and correspondingly reduce the decision-making efforts. It can be concluded that non-choice deferral behavior makes consumers have a lower level of hopefulness. Accordingly, hypothesis H2 is proposed.

H2: Consumers’ choice deferral behavior significantly impacts their level of hopefulness. Non-choice deferral behavior can reduce consumers’ level of hopefulness, while choice deferral behavior can improve consumers’ level of hopefulness.

Emotional states can influence decision-makers’ decision-making behavior. According to the broaden-and-build theory of positive emotions, positive emotions can expand a person’s instantaneous cognition and action abilities and broaden the scope of a person’s attention, cognition, and action (Fredrickson, 1998). Individuals in a positive emotional state can view problems from a broader perspective with a more positive attitude and more meaning, thus fully mobilizing their internal motivation to achieve goals. Conversely, if individuals are in a negative emotional state, they view problems with narrower sight and focus on a problem’s negative aspects, which is not conducive to achieving targets (Zhang and Wang, 2017). In addition, one’s emotional state can further influence decision-making results by influencing decision-making behavior. One study found that different emotional valences had varying effects on individuals’ time discount rate; prior to decision-making, positive emotions can reduce the time discount rate, while negative emotions can increase the time discount rate (Jiang and Sun, 2019). According to this analysis, since hopefulness is a special positive emotional state, we can conclude that higher hopefulness can make consumers more open-minded, care about the future, and more inclined to LL. Relatively, lower hopefulness narrows consumers’ minds, causes them to concentrate on the present, and tend toward sooner-smaller interests more often. Hence, hypothesis H3 is proposed.

H3: Consumers’ level of hopefulness can significantly affect their intertemporal choice preference. Higher levels of hopefulness cause consumers to prefer LL; lower levels of hopefulness cause consumers to prefer SS.

When we synthesize H1, H2, and H3, we find that choice deferral gives consumers more time to think and act, reduces their negative emotions effectively, and causes their emotions to change for the positive. To optimize decision-making results, consumers are willing to think actively as well as work to discover more solutions—i.e., they have higher dynamic thought and path thought. According to the definition of hopefulness, choice deferral does give consumers a higher level of hopefulness. Simultaneously, according to the broaden-and-build theory of positive emotions, we learn that higher levels of hopefulness can broaden consumers’ thoughts, give them a longer-term vision, and help them analyze and solve problems more rationally. Therefore, when given a choice of interests, consumers will pursue long-term interests first, and then, prefer LL in intertemporal choice making. Comparatively, non-choice deferrals leave consumers with insufficient time to think and makes them reluctant to think comprehensively in a short amount of time, which means consumers have lower levels of hopefulness. As a result, lower levels of hopefulness can cause consumers to feel negative and focus only on immediate interests, which in turn means that they are unable to fully consider a purchase, are more irrational and impulsive, and will prefer sooner-smaller interests (SS). Based on these reflections, hypothesis H4 is proposed.

H4: Consumers’ level of hopefulness plays a mediating role in the relationship between choice deferral behavior and intertemporal choice preference.

Although consumers’ hopefulness levels can affect their intertemporal choice preferences as well as the relationship between choice deferral behavior and intertemporal choice preference, this effect may vary with the degree of information integrity consumers understand and perceive. The integrity of perceived information is one of the important factors affecting consumer decision-making, information overload and lack of information will both affect individual value judgment of options (Afzal et al., 2008). According to the prospect theory, people detest risks and losses, and they are more averse to losses than gains (Lin et al., 2019). Some studies show that if residents are uncertain about their income, it has a significant effect on their consumption—the higher the uncertainty, the more reluctant they are to consume (Feng, 2019). As aforementioned, the higher the level of consumer hopefulness, the more positive and rational consumers are, and the more likely consumers are to obtain further information to optimize their decisions, which influences them to prefer LL in intertemporal choice. When we combine this preference with the prospect theory, we believe that if consumers perceive a lower level of information integrity, they will perceive greater risk. As a result, they will spend more time retrieving and processing information in order to avoid losing interests, which enhances the level of impact hopefulness has on intertemporal choice preference—i.e., they prefer LL. However, if consumers perceive more complete information, they are more confident in decision-making. Even if the level of hopefulness is high, consumers will not consume additional energy obtaining information but are more likely to use the information they have mastered to make decisions. Therefore, the level of impact of hopefulness on intertemporal choice preference is no longer significant. As aforementioned, the lower the level of consumer hopefulness, the more negative their mood; the lower the motivation to optimize decision-making, the less willing consumers are to make efforts to optimize decision-making. As a result, these consumers prefer shorter-smaller interests (SS) in intertemporal choice making. If, at this juncture, consumers perceive lower degrees of information integrity, it will further reduce their decision-making confidence, enhance their negative emotions, and weaken their motivation to optimize decision-making. All of these factors enhance the impact of the level of consumer hopefulness on intertemporal choice preference—i.e., they prefer shorter-smaller interests (SS). However, if consumers perceive a high level of information integrity, they will more confidently make satisfactory decisions. Even if they are not willing to continue spending their energy on seeking new information, they will make choices based on existing information. At this time, consumer hopefulness levels will not significantly impact their intertemporal choice preferences. Based on the prospect theory, hypothesis H5 is proposed.

H5: Consumers’ perceived information integrity can moderate hopefulness’ fully mediating role between choice deferral behavior and intertemporal choice preference.

**FIGURE 3:**
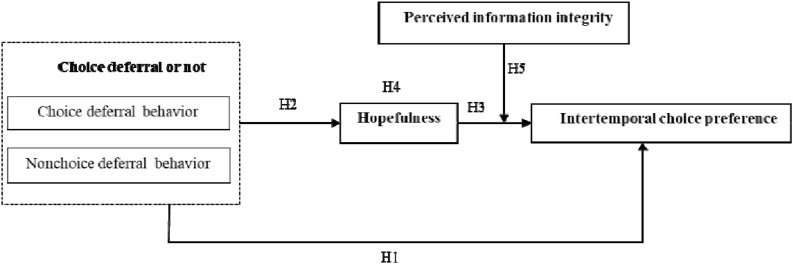
Research model.

Based on the literature review and our research hypotheses, a research model is proposed and shown in Figure 3.

## Materials and Methods

### Participants

In this study, a questionnaire survey was conducted among the staff of a university in Guangxi, China. In the pre-experiment, 20 staff were randomly invited to conduct a questionnaire survey. A total of 20 questionnaires were distributed and 20 valid questionnaires were collected. In the formal experiment, 240 staff were randomly invited to conduct a questionnaire survey, and a total of 240 questionnaires were distributed. After deleting the questionnaires with problems such as random filling and inconsistent logic, 175 valid questionnaires were finally returned. In the formal experiment, the proportion of male and female subjects was balanced (52% males; 48% females). The age of subjects was mainly from 18 to 30 years old (94%), the occupation was mainly students (84%), and the education was mainly undergraduates (58%). Table 1 shows the composition of these valid samples.

**TABLE 1:** Composition of valid samples.

Items	Category	Number	Proportion
Sex	Male	91	52%
	Female	84	48%
Age	<18	0	0
	18–30	165	94%
	31–45	2	1%
	>45	8	5%
Qualification	Under university	12	7%
	Undergraduate	101	58%
	Graduate or above	62	35%
Profession	Government or institution	8	5%
	Enterprise	13	7%
	Student	147	84%
	Self-employed	6	3.4%
	Retired	1	0. 6%

### Procedure

#### Pre-experiment

The purpose of the pre-experiment is to select the type of products to be used in the formal experiment through a questionnaire survey. The main question of the questionnaire is for what kind of goods (such as food, electronic products, fitness cards, clothing, etc.) the subjects will defer their choice and the reasons. Finally, the artificial intelligence box of virtual brand is selected as the experimental material.

#### Formal Experiment

The purpose of the formal experiment is to collect the required data by issuing questionnaires to explore the relationship between variables. Two different questionnaires were developed based on whether to delay selection. Each questionnaire covered all the variables to be measured. When sending out the questionnaire, it was first explained to the subjects that the purpose of this time is to collect data for academic research rather than for commercial purposes. In addition, the questionnaire is anonymous and does not involve personal privacy. Then, random questionnaires were sent to the subjects to fill in and answer. The first part of the questionnaire is demographic information, and the second part is the items of measurement variables. Finally, after the subjects completed the questionnaire, they expressed their thanks.

### Measures

#### Choice Deferral Behavior (Independent Variable)

In the scenario design of the questionnaire, the questionnaire will be pre-set to be filled in under the two scenarios of choice deferral behavior/non-choice deferral behavior, and the subjects will be divided into two groups accordingly for the control experiment. The non-choice deferral behavior scenario is described as follows: “if you need to decide whether to buy it now, how would you think? Please answer the following questions according to your personal feelings.” The scenario of choice deferral behavior is described as follows: “now you don’t need to decide whether to buy immediately, we will contact you in a week, and then you will make a purchase decision, during which time you will have enough time to consider.”

#### Perceived Information Integrity (Moderator)

It is a continuous variable. When measuring, the subjects are shown the product information list prepared in advance. The list is made by referring to the official attribute information of the product, but the brand of the product is not disclosed to avoid the interference of the brand. Then, the definition of perceived information integrity was given to the subjects: “the quantity and comprehensiveness of information meet the needs of your purchase decision.” Then tell the subjects: “the contents in the Table are all the product information you have obtained. Please rate the information integrity you have perceived. The larger the number, the more complete it is.” Finally, consumers were asked to score with the Likert Scale 5.

#### Hopefulness (Mediation)

It is a continuous variable, referring to the State Hope Scale developed by Snyder et al. (1996). To appropriately measure subjects’ hopefulness levels, we modify the scale based on this study’s content. The State Hope Scale is divided into two dimensions—dynamic thought and path thought. Each dimension includes three items, so the scale contains six items in total. Moreover, the scale is scored by Likert Scale 8, the higher the score, the higher the hopefulness level. The items of the scale are as follows: “I will consider different factors to decide whether to buy or not,” “I hope I can make a satisfactory choice,” “I will consider various ways or methods to help me make a choice,” “I am willing to actively consider,” “I will consider from different angles,” “I believe I can make a satisfactory choice.”

#### Intertemporal Choice Preference (Dependent Variable)

There are many measurement models, such as hyperbolic discount model, quasi-hyperbolic discount model, the delay function (Cruz Rambaud and González Fernández, 2020), and new indicators (Cruz Rambaud and González Fernández, 2019). The measurement index used in this paper is time discount rate, which is a continuous variable. Decision-makers discount utility at different points, according to a certain ratio. The total discounted utility determines their preference in intertemporal choice making, and the ratio is the time discount rate (Zhang et al., 2018). Based on the amount of money subjects are willing to pay for a product 1 month later, we use Yang et al.’s (2018) calculation method, the hyperbolic discount model, which calculates the time discount rate of subjects. The smaller the time discount rate, the more often LL are preferred; the larger the time discount rate, the more often sooner-smaller interests (SS) are preferred. The formula of the hyperbolic discount model is V = A/(1 + KD), where V is the current amount, A is the future amount, D is the delay time (a unit is equal to 1 day), and K is the time discount rate (Loewenstein and Prelec, 1992). The expression of this item is as follows: “How much are you willing to spend on it in 3 months, compared with spending 299 Yuan on it now and starting to use it?”. In order to eliminate the influence of other factors in the extended period of time, consumers are also asked to fill in the amount directly in the delayed situation.

### Data Analyses

SPSS was used for data analysis. Firstly, Pearson correlation analysis was used to test the relationship between variables, and then the mediating analysis and moderated mediating effect analysis were carried out twice to ensure the reliability of the experimental results. The first time is to use SPSS for traditional hierarchical regression analysis, and the second time is to use macro process of SPSS to verify the mediating effect and moderated mediating effect through the bootstrap method (Hayes, 2013, 2015). The bootstrap method is widely used in international top journals (Du et al., 2020; Quan et al., 2020).

## Results

### Reliability Analysis

The State Hope Scale’s Cronbach’s alpha coefficient is 0.88. The scale is divided into two dimensions—path thought and dynamic thought. The Cronbach’s alpha coefficient of path thought is 0.89, and the Cronbach’s alpha coefficient of dynamic thought is 0.70. All of the coefficient scores are greater than 0.7, showing that the scale has good reliability and good internal consistency.

### Validity Analysis

Since only the mature scale was used to measure the level of hopefulness in the questionnaire, the validity of the scale (six items) was analyzed. The maturity scale was adapted to make it more suitable for the research situation, and it was checked by experienced experts and scholars, which can be regarded as good content validity. By confirmatory factor analysis with Amos, the AVE = 0.56 and CR = 0.88, respectively, indicating that the scale has good structural validity. Through the above analysis, the scale has good validity.

### Control of Common Method Biases

First, to ensure procedure control, the order, language expression, and layout design of the questionnaire items are revised and perfected repeatedly. Additionally, an anonymous survey is adopted, and questionnaires are distributed and collected at different times and places, which, in part, controls common method biases. Second, to ensure statistical control, Harman’s one factor test is used. Exploratory factor analysis was used to examine the four variables: choice deferral behavior, level of hopefulness, degree of perceived information integrity, and time discount rate. The results show that the KMO value is 0.52, and the significance level of Bartlett’s test is 0.000. The first common factor explains 49.96% of the total variance, which does not exceed 50%. Therefore, no serious problem exists among common method biases.

### Variables’ Correlation Description

Table 2 shows the correlation coefficient matrix among variables. It can be seen from Table 2 that the choice deferral behavior is positively correlated with the time discount rate, and negatively correlated with the level of hopefulness. The time discount rate is negatively correlated with the level of hopefulness, which is consistent with the hypothesis proposed in the previous paper, and lays the foundation for the subsequent test of the relationship between them.

**TABLE 2:** Correlation among variables.

Variable	Time discount rate	Hopefulness	Choice deferral behavior (deferraland non-deferral)	Perceived information integrity
Time discount rate	—			
Hopefulness	–0.41^**^	—		
Choice deferral behavior (deferral & non-deferral)	0.16^*^	–0.47^**^	—	
Perceived information integrity	–0.24^**^	0.55^**^	0.06	—

***P < 0.001, **P < 0.01, *P < 0.05.

Testing the relationship between choice deferral behavior, hopefulness, intertemporal choice preference and perceived information integrity.

SPSS was used to conduct hierarchical regression on choice deferral behavior, level of hopefulness, intertemporal choice preference and perceived information integrity. The results are shown in Table 3.

**TABLE 3:** Analysis of the results of hierarchical regression.

Variable	Hopefulness		Time discount rate					
	M2	M6	M1	M3	M4	M5	M7	M8
Choice deferral behavior	0.46	0.44^**^	–0.16^*^		0.04	–0.10	0.04	0.05
Hopefulness				–0.41*	–0.43^*^		–0.42	–0.25
Perceived Information integrity		0.56				–0.20	–0.01	–0.11
Perceived information integrity* Hopefulness								–0.40^***^
*R*^2^	0.21	0.49	0.03	0.17	0.17	0.08	0.17	0.31
*F*	48.1	83.78	4.64^*^	35.23	17.66	7.60	11.71	18.86***
ΔR^2^	0.21	0.49	0.02	0.17	0.16	0.07	0.16	0.29

“M” is the abbreviation of “model”; ***P < 0.001, **P < 0.01, *P < 0.05.

The regression analysis of the time discount rate and choice deferral behavior forms model 1, the result shows that choice deferral behavior is significantly correlated with the time discount rate (β = –0.16, *p* < 0.05). Therefore, compared with non-choice deferral behavior, choice deferral behavior can reduce consumers’ time discount rate and make them prefer LL. Hence, H1 is supported. Regression analysis of hopefulness and choice deferral behavior forms model 2, and the result shows that choice deferral behavior is significantly correlated with hopefulness (β = 0.47, *p* < 0.001). Thus, compared with non-choice deferral behavior, choice deferral behavior can improve consumers’ hopefulness. Therefore, H2 is supported. Regression analysis of the time discount rate and hopefulness forms model 3, and the result shows that hopefulness is significantly correlated with the time discount rate (β = –0.41, *p* < 0.000). Therefore, the higher the hopefulness, the smaller the consumers’ time discount rate, and the more consumers prefer LL. As a result, H3 is supported.

### Mediating Effect Test

First, this paper uses SPSS and Wen and Ye (2014) method to test the mediating effect of hopefulness level. From Table 3, we see that: (1) the independent variable (choice deferral behavior) has a significant effect on the dependent variable (time discount rate) (M1: β = –0.16, *p* < 0.05); (2) the independent variable (choice deferral behavior) has a significant effect on the mediation variable (hopefulness) (M2: β = 0.47, *p* < 0.001); (3) the independent variable (choice deferral behavior) and the mediation variable (hopefulness) have a significant effect on the dependent variable (time discount rate). Hopefulness has a significant effect on the time discount rate (M4: β = –0.43, *p* < 0.000), while choice deferral behavior has no significant effect on the time discount rate (M4: β = 0.04, *p* > 0.05). Therefore, hopefulness is a complete mediator and H4 is supported.

Using the macro process of SPSS and bootstrap method, model 4 was selected and 5000 samples were repeatedly selected to verify the mediating effect (Hayes, 2013, 2015), the specific results are shown in Table 4. Choice deferral behavior’s direct effect on intertemporal choice preference is –0.11, the 95% confidence interval is [–0.52, 0.31], including 0, showing that the direct effect is not significant. The indirect effect of choice deferral behavior on intertemporal choice preference is 0.54 with a 95% confidence interval [0.15, 1.04], excluding 0, revealing that the indirect effect is significant. The total effect of choice deferral behavior, hopefulness and intertemporal choice preference is 0.43, the 95% confidence interval is [0.12, 0.83], excluding 0, proving that the total effect is significant. This proves the complete mediation effect of hopefulness.

**TABLE 4:** Mediating effect.

Type of effect	Effect value	Boot SE	Bootstrap 95%CI		Relative effect
			Low limit	High limit	
Total effect	0.43	0.18	0.12	0.83	1
Direct effect	–0.11	0.21	–0.52	0.31	–0.24
Indirect effect	0.54	0.23	0.15	1.04	1.24

### Moderated Mediating Effect

First, this paper uses SPSS to test the moderating mediating effect by referring to the methods of Liu and Li (2016). From Table 3 we learn that: (1) time discount rate regressions show that the choice deferral behavior coefficient is significant (M5: β = –0.15, *p* < 0.005); (2) hopefulness, choice deferral behavior, and perceived information integrity regressions show that the choice deferral behavior coefficient is significant (M6: β = 0.44, *p* < 0.001); (3) time discount rate, choice deferral behavior, perceived information integrity and hopefulness regressions show that the coefficient of hopefulness is significant (M7: β = –0.42, *p* < 0.001); (4) time discount rate, choice deferral behavior, perceived information integrity, hopefulness, and the interaction between perceived information integrity and hopefulness regressions show that the interaction item coefficient is significant (M8:β = –0.40, *p* < 0.001). The above test steps fully meet the moderated mediating effect test criteria, which shows that perceived information integrity has a significant moderating effect on hopefulness’ mediating effect. Therefore, H5 is supported.

**FIGURE 4:**
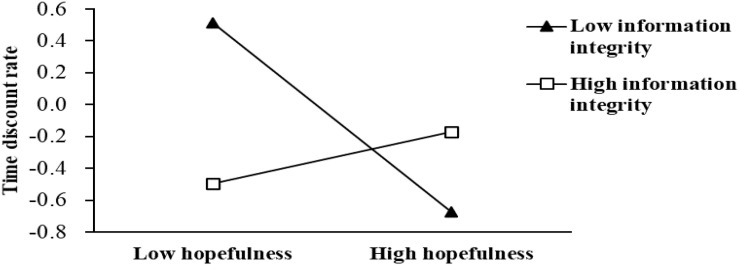
Moderating effect diagram.

Secondly, in order to ensure the reliability of the conclusion, the macro process of SPSS is used to verify the moderated mediating effect again by the bootstrap method, selecting model 14 and repeatedly sampling 5000 samples (Hayes, 2013, 2015). Among them, the grouping of regulatory variables is based on the average plus or minus a standard deviation (Wang et al., 2017; Lian et al., 2018). The specific results are shown in Table 5. Using grouping, a simple slope map was created by either adding or subtracting a standard deviation from the mean of perceived information integrity (see Table 5). Table 5 shows that when perceived information integrity is high, the 95% confidence interval is [–0.03, 0.09], including 0, proving that the moderating effect is not significant. In contrast, when perceived information integrity is low, the 95% confidence interval is [–0.18, –0.10], excluding 0, showing that the moderating effect is significant. When perceived information integrity decreases, the negative effect of hopefulness on the time discount rate is expected to increase. Figure 4 more intuitively reflects the moderating role of perceived information integrity.

**TABLE 5:** Moderated mediating effect.

	Perceived information integrity	Effect	*SE*	*T*	*p*	LLCI	ULCI
M-SD	–1.17	–0.14	0.02	–6.50	0.00	–0.18	–0.10
M	0.00	–0.06	0.02	–2.61	0.01	–0.10	–0.01
M + SD	1.17	0.03	0.03	1.02	0.31	–0.03	0.09

## Discussion

This paper studies the influence of consumers’ choice deferral behavior on their intertemporal choice preference, and verifies the research model through questionnaire survey and data analysis. It is found that consumers’ choice deferral behavior can have a significant impact on their intertemporal choice preference, and the level of hopefulness plays a mediating role in the relationship between them. Compared with non-choice deferral behavior, consumers are more willing to make more efforts to make decisions after a delayed choice, and they will optimize their decisions through different paths and methods. That is to say, choice deferral behavior makes consumers have a higher level of hopefulness, so they prefer LL in intertemporal decision-making. The degree of information integrity perceived by consumers will have a moderating effect on the relationship between the level of hopefulness and intertemporal decision-making. When consumers perceive that they have incomplete information, they can also perceive higher risks (Li and Huang, 2016). Because most people are loss averse (Lin et al., 2019), consumers are more willing to take measures to optimize decisions to avoid losses. This will make consumers focus on the overall situation, pay attention to the nature of the problem, and thus prefer LL in intertemporal decision-making. On the contrary, when consumers perceive that the information is relatively complete, they will rely on the existing information for decision-making and ignore some details (Evans, 2008), which will also lead to their narrow vision and only focus on a part of things, so that they prefer SS in intertemporal decision-making. In other words, the relationship between the level of hopefulness and intertemporal choice preference will be regulated by the perceived information integrity, and then the mediating effect of choice deferral behavior on intertemporal choice preference through the level of hopefulness will also be regulated by the perceived information integrity. These contents are verified by experiments and supported.

### Theoretical Contributions

Firstly, based on the construal level theory, this paper reveals the influence mechanism of choice deferral behavior on intertemporal choice preference, and deepens the research on the after-effect of choice deferral behavior. Because consumers may lose sales opportunities if they do not make a purchase decision immediately, previous scholars pay more attention to the factors that lead to consumers’ choice deferral behavior, such as different decision-making scenarios (Huang and Wang, 2019), the attractiveness of options (Larasati and Yeh, 2016), individual tolerance of uncertainty (Huang et al., 2014), and so on, so as to reduce this behavior as much as possible. However, the process of shopping is dynamic and relevant, consumers may not give up their purchase after a delayed choice, they may still consider the purchase decision, and it may even affect their future purchase decisions. Therefore, it is necessary to understand the change process of consumers’ shopping preferences after choice deferral behavior. It is found that consumers’ choice deferral behavior does have an impact on their intertemporal choice preference, and choice deferral behavior will make consumers prefer LL in intertemporal decision-making. Therefore, this study has greatly expanded the study on the after-effect of choice deferral behavior.

Secondly, based on the broaden-and-build theory of positive emotions and the prospect theory, this study explored the mediating role of hopefulness and the moderating role of perceived information integrity. Emotion is one of the important factors that affect individuals’ choice deferral behavior and intertemporal choice preference, scholars have done a lot of research, but few scholars pay attention to hopefulness (Wang and Liu, 2009; Guan et al., 2015). Based on the previous conclusions, this paper studies the relationship among hopefulness, choice deferral behavior, and intertemporal choice preference, and finds that the level of hopefulness plays a mediating role between choice deferral behavior and intertemporal choice preference. In addition, information is one of the bases of an individual’s ability to make decisions (Jacoby et al., 1978), and many scholars have studied its impact on individual choice deferral behavior and intertemporal choice preference from different perspectives. Some scholars have found that information presentation form and individual specific knowledge level can affect individual’s choice deferral behavior (Lange and Krahe, 2014), and others have found that an individual’s information processing process can affect their intertemporal decision preference (Sun and Jiang, 2016). This paper innovatively analyzes the relationship of consumers’ perceived information integrity, choice deferral behavior and intertemporal choice preference. It was found that consumers’ perceived information completeness has a moderating effect between their hopefulness and intertemporal choice preference, as well as can further affect the mediating effect of hopefulness between choice deferral behavior and intertemporal choice preference, thus forming a moderated mediating model.

Thirdly, this study explored the positive role of choice deferral behavior. Most scholars believe that consumers’ choice deferral behavior means missing sales opportunities for businesses, so it is usually regarded as a negative factor (Kunter and Ross, 2009; Godinho et al., 2016), but this paper believes that consumers’ choice deferral behavior does not have an absolutely negative impact on businesses. Consumers make delayed choices in order to obtain more information, make better decisions, and avoid regret for wrong decisions in the future (Bastardi and Eldar, 1998; Cooke et al., 2001). Although it does not bring immediate benefits for businesses, it can promote businesses to improve product quality and service, improve marketing strategies, etc., which is conducive to the long-term development of businesses. From this point of view, consumers’ choice deferral behavior is beneficial to themselves and businesses. This paper provides a new and dialectical perspective for the study of consumers’ choice deferral behavior. In the future, we need to explore more perspectives to study the choice deferral behavior.

### Practical Implications

First, businesses can adopt different marketing methods according to consumers’ different purchase behaviors (non-choice deferral/choice deferral). According to the previous research, non-choice deferral behavior makes consumers prefer SS, while choice deferral behavior makes consumers prefer LL. Therefore, if consumers choose whether to buy immediately, it means that consumers pay more attention to the current interests. In marketing, businesses should focus on showing consumers the benefits of current purchase, such as being able to catch up with the trend and use the product immediately. If consumers decide to postpone the choice, or have considered it for a period of time, it means that consumers pay more attention to the long-term interests, then in marketing, businesses need to highlight the cost performance of products, after-sales protection, and other aspects.

Second, businesses can use differentiated marketing methods according to the level of consumers’ sense of hopefulness. As can be seen from the previous article, hopefulness includes two aspects: dynamic path and thinking path. The higher the level of hopefulness, the more consumers prefer LL. Therefore, businesses can first communicate with consumers to know their sense of hopefulness, such as whether consumers are willing to learn more about product information and whether they will compare different products through multiple channels. When the level of consumers’ sense of hopefulness is high, it indicates that they are willing and will make decision-making efforts, then businesses can carry out marketing from more rational aspects, such as highlighting the material, composition and production technology of products. And when the level of consumers’ sense of hopefulness is low, it indicates that they are not willing and will not make too much decision-making effort, then businesses can carry out marketing from more emotional aspects, such as emphasizing the products’ packaging, spokesperson, etc.

Third, businesses can carry out marketing according to the degree of information integrity perceived by consumers. The results show that when consumers perceive incomplete information, they perceive higher risk, and the level of their sense of hopefulness has a strong effect on intertemporal decision preference. In marketing, businesses need to know whether consumers think they have enough information. If consumers think it is useless to have enough information, businesses should actively provide some official, professional, and authoritative information, which can also reflect the professionalism and sincerity of businesses.

### Limitations and Future Research

First of all, this paper mainly compares the changes of consumers’ intertemporal choice preference under two different behaviors of choice deferral and non-choice deferral, and does not further study whether the length of choice delay can affect consumers’ intertemporal choice preference. In the future, we can design different delay times to carry out further research on the basis of this study. Secondly, the subjects of this study are mainly Chinese college students. In the future, this paper can be used as a research paradigm to select more diverse groups, such as groups from different countries and different income levels, to compare the impact of different types of groups’ choice deferral behavior on their intertemporal choice preferences. Finally, more factors need to be included in the research model, such as product type, consumer income, and other factors. Whether they affect the relationship between consumers’ choice deferral behavior and intertemporal choice preference also needs to be explored, so as to make the research better adapt to practical application in different scenarios.

## Data Availability Statement

The raw data supporting the conclusions of this article will be made available by the authors, without undue reservation.

## Ethics Statement

The studies involving human participants were reviewed and approved by Guangxi University of Ethics Committee. Written informed consent was not required to participate in this study in accordance with the national legislation and the institutional requirements.

## Author Contributions

H-LW, C-YH, S-YZ, and BL: study conception and design. H-LW, C-YH, and BL: acquisition of data. H-LW and C-YH: analysis and interpretation of data and drafting of the manuscript. All authors contributed to the article and approved the submitted version.

## Conflict of Interest

The authors declare that the research was conducted in the absence of any commercial or financial relationships that could be construed as a potential conflict of interest.
